# Gellan gum-gelatin based cardiac models support formation of cellular networks and functional cardiomyocytes

**DOI:** 10.1007/s10616-024-00630-5

**Published:** 2024-05-02

**Authors:** Hanna Vuorenpää, Joona Valtonen, Kirsi Penttinen, Sanna Koskimäki, Emma Hovinen, Antti Ahola, Christine Gering, Jenny Parraga, Minna Kelloniemi, Jari Hyttinen, Minna Kellomäki, Katriina Aalto-Setälä, Susanna Miettinen, Mari Pekkanen-Mattila

**Affiliations:** 1 Adult Stem Cell Group, Faculty of Medicine and Health Technology, Tampere University, Tampere, Finland; 2 Tays Research Services, Wellbeing Services County of Pirkanmaa, Tampere University Hospital, Tampere, Finland; 3 Heart Group, Faculty of Medicine and Health Technology, Tampere University, Tampere, Finland; 4 Computational Biophysics and Imaging Group, Faculty of Medicine and Health Technology, Tampere University, Tampere, Finland; 5 Biomaterials and Tissue Engineering Group, Faculty of Medicine and Health Technology, Tampere University, Tampere, Finland; 6 Department of Plastic and Reconstructive Surgery, Tampere University Hospital, Tampere, Finland; 7 Heart Hospital, Tampere University Hospital, Tampere, Finland

**Keywords:** Cardiomyocytes, Hydrogels, Vasculature, Mesenchymal stem cells, Electrophysiology, In vitro model

## Abstract

Cardiovascular diseases remain as the most common cause of death worldwide. To reveal the underlying mechanisms in varying cardiovascular diseases, in vitro models with cells and supportive biomaterial can be designed to recapitulate the essential components of human heart. In this study, we analyzed whether 3D co-culture of cardiomyocytes (CM) with vascular network and with adipose tissue-derived mesenchymal stem/stromal cells (ASC) can support CM functionality. CM were cultured with either endothelial cells (EC) and ASC or with only ASC in hydrazide-modified gelatin and oxidized gellan gum hybrid hydrogel to form cardiovascular multiculture and myocardial co-culture, respectively. We studied functional characteristics of CM in two different cellular set-ups and analyzed vascular network formation, cellular morphology and orientation. The results showed that gellan gum-gelatin hydrogel supports formation of two different cellular networks and functional CM. We detected formation of a modest vascular network in cardiovascular multiculture and extensive ASC-derived alpha smooth muscle actin -positive cellular network in multi- and co-culture. iPSC-CM showed elongated morphology, partly aligned orientation with the formed networks and presented normal calcium transients, beating rates, and contraction and relaxation behavior in both setups. These 3D cardiac models provide promising platforms to study (patho) physiological mechanisms of cardiovascular diseases.

**Supplementary Information:**

The online version contains supplementary material available at 10.1007/s10616-024-00630-5.

## Introduction

Cardiovascular diseases remain as the principal cause of death worldwide. Neither basic research nor drug development has fully responded to the increasing health care demand by providing safe and efficient treatments for patients suffering from different types of cardiovascular diseases. In drug development, lack of relevant preclinical test systems in predicting human effects, beside time- and cost intensity, is complicating the development of new treatments (Eder et al. 2016). Induced pluripotent stem cell technology offers an attractive source of human cells not only for drug development but also for tissue engineering and regenerative medicine applications. The integration of patient-specific induced pluripotent stem cell derived cardiomyocytes (iPSC-CM) allows easy generation of tissue-like preparations and modelling of patient-specific genetic mutations (Giacomelli et al. 2017).

Successful cardiac in vitro modeling requires CM interactions with other cell types naturally present in the heart. Transcriptomic study of adult human heart cellular composition revealed eleven main cell types including atrial and ventricular cardiomyocytes, fibroblasts, endothelial cells, pericytes, smooth muscle cells, myeloid and lymphoid immune cells, adipocytes, mesothelial cells and neuronal cells (Litviňuková et al. 2020). Furthermore, dynamic interactions between cells and ECM allow normal cardiac structure and function. While cardiomyocytes form the largest cell population in the heart, pericytes and endothelial cells are abundant especially in the myocardial blood vessels. (Howard and Baudino 2014) In traditional 2D in vitro models, the characteristic mechanisms and architecture of cardiovascular system are difficult to recapitulate. Encapsulating cells in 3D scaffolds can provide mechanical stimulus and topography required by CM, reflecting the physiological tissue microenvironment more precisely than planar, stiff cell cultures surfaces (Rodrigues et al. 2018; Tulloch et al. 2011). Hydrogels provide an attractive option to mimic in vivo microenvironment and can be modified by selecting suitable biomaterials, crosslinking methods and fabrication strategies to support cardiac functions (Li et al. 2018). Indeed, hydrogels are the most widely used polymers in cardiac tissue engineering as they enable simultaneous gelation of cells and the polymer, promote cell assembly as well as subsequent functionality and maturation of CM (Tenreiro et al. 2021). Earlier, we applied hydrazone chemistry to combine gelatin and gellan gum biopolymers for 3D culture of iPSC-CM (Koivisto et al. 2019). We demonstrated that iPSC-CM remained viable and functional when plated either on top or when embedded inside the hydrogel (Koivisto et al. 2019).

Insufficient iPSC-CM maturation forms a bottleneck that hampers the use of human iPSC-CM in in vitro disease modeling, drug development and in regenerative medicine. Cardiomyocyte maturation refers to developmental changes in cell structure, metabolism, function, and gene expression that drives fetal CM into adult CM phenotype (Guo and Pu 2020). Currently, there is widely acknowledged gap between native CM and iPSC-CM development in vitro and, despite several efforts in improving maturity with engineering approaches, iPSC-CM cultures so far poorly capture all characteristics of native CM (Tenreiro et al. 2021). Adult CM exhibit a rod shape morphology whereas iPSC-CM are typically round- or triangular- shaped and require physical cues to adopt native morphology (Gerdes et al. 1992; Guo and Pu 2020). Force-generation of iPSC-CM is accelerated during cardiac maturation as sarcomeric structures becomes longer and more organized (Bird et al. 2003; Lundy et al. 2013), thus improving the contractility. The coordinated and strong contraction of multiple sarcomeres is achieved in adult CM (Scuderi and Butcher 2017; Steinhoff et al. 2017), which hold more developed calcium handling machinery compared to iPSC-CM with slow excitation–contraction coupling and reliance on l-type channels for the Ca^2+^ increase (Bers 2002).

Several cardiac in vitro models focus on assessment of efficacy and cardiotoxicity that are major bottlenecks in development of new drugs. A commonly used in vitro preclinical test system employs SCN5A-encoded HEK293 non-myocyte cells. Compared to HEK293 cells, hiPSC-CM have been shown to efficiently predict the effects of drugs on cardiac action potential, voltage-dependent Na^+^ channels and, importantly, hERG coded potassium channel (Lee et al. 2018). For a while, 3D CM monocultures have been proposed as an improved test system in predicting human cardiac effects (Chaudhari et al. 2016; Lu et al. 2017; Takeda et al. 2018). However, these test platforms often lack the essential cell–cell interactions and paracrine signaling between CM and other cell types that regulate normal heart functions in vivo (Howard and Baudino 2014; Giacomelli et al. 2020).

There are several reports on the benefits of not only EC co-culture with CM (Brutsaert 2003; Narmoneva et al. 2004; Caspi et al. 2007) but on vascularizing cardiac in vitro models (Caspi et al. 2007; Vuorenpää et al. 2014, 2017; Koivisto et al. 2022; King et al. 2022). Earlier, we have shown that combining vascular-like network with CM in 2D platform results in upregulation of genes related to CM differentiation and maturation and in vivo-like morphology of cardiomyocytes (Vuorenpää et al. 2014, 2017). In the present study, we introduced 3D environment with biocompatible hydrogel and analyzed whether vascularization or co-culture with adipose tissue-derived mesenchymal stem/stromal cells (ASC) is beneficial in supporting CM functionality. CM were cultured with either endothelial cells (EC) and ASC or with ASC alone in hydrazide-modified gelatin and oxidized gellan gum hybrid hydrogel to form cardiovascular multiculture and myocardial co-culture, respectively. We analyzed CM functionality using video microscopy and calcium imaging and morphology of CM and vascular network in structural level. Our results demonstrate that both cardiac models supported CM functionality and can, therefore, be used as novel methods to study cardiovascular (patho)physiology.

## Materials and methods

### Ethical consideration

This study conforms to the principles outlined in the Declaration of Helsinki. The use of human iPSC was approved by Ethics Committee of the Pirkanmaa Hospital District, Tampere, Finland (Approval Number R08070). Written informed consents were received from donors. Human ASC were isolated from subcutaneous tissue samples that were received from three different donors (donor information on Table S1). Adipose tissue samples were obtained at the Tampere University Hospital Department of Plastic Surgery with the donor's written informed consent and processed under ethical approval of the Ethics Committee of the Pirkanmaa Hospital District, Tampere, Finland. (Approval Numbers R15161).

### Cell isolation, differentiation, and culture

#### Isolation and culture of human adipose tissue derived stem/stromal cells

The cells were isolated as described previously (Kyllönen et al. 2013). Briefly, adipose tissue samples were cut into smaller pieces and digested with collagenase type I (1.5 mg/ml, Thermo Scientific) for 60–90 min at + 37 ℃. After centrifugation, red blood cells were lysed with water and the cells were centrifuged and filtered. The ASC were cultured in α-MEM (Thermo Scientific) supplemented with 5% human serum (HS, Serana) and 100 U/mL penicillin, and 100 μg/mL streptomycin (Lonza), expanded for 3–5 days and used in passages 1 or 4. For ASC characterization, plastic adherence and surface marker expression of the used cell lines was studied to identify the mesenchymal origin of ASC using flow cytometry (Table S2). The identification criteria is defined by the International Society for Cellular Therapy (Dominici et al. 2006; Bourin et al. 2013).

#### Culture of human umbilical vein endothelial cells

Pooled human umbilical vein endothelial cells (HUVEC) expressing green fluorescent protein (GFP) were commercially obtained from Cellworks. GFP-HUVEC were cultured as previously described (Mykuliak et al. 2022), expanded for 3–5 days and used in passages 3–5.

#### Generation of induced pluripotent stem cell line and cell culture of pluripotent stem cells

Establishment of patient-specific iPSC line UTA.04602.WT from healthy individual has been described earlier (Takahashi et al. 2007) and the results of the cell line characterization has been presented earlier (Lahti et al. 2012). UTA.04602.WT cells were cultured on mitomycin C inactivated mouse embryonic fibroblasts (MEF) in KSR medium consisting of DMEM/F-12 (Invitrogen) supplemented with 20% KnockOut serum replacement (Invitrogen), 1% non-essential amino acids (NEAA, Lonza Group Ltd, Basel, Switzerland), 2 mM Glutamax (Invitrogen), 50 U/ml penicillin/streptomycin (Lonza Group Ltd, Basel, Switzerland), 0.1 mM β-mercaptoethanol (Invitrogen) and 7.8 ng/ml basic fibroblast growth factor (R&D Systems). The medium was refreshed daily and using 1 mg/ml collagenase IV (Invitrogen) cell colonies were passaged onto a new MEF layer once a week.

#### Differentiation of induced pluripotent stem cell derived cardiomyocytes

Human iPSC were differentiated into cardiomyocytes with small molecule differentiation method based on Karakikes et al. (2014) protocol as described previously (Prajapati et al. 2021). Briefly, the hiPSCs were treated with versene, resuspended to mTeSR1 medium supplemented with 5 µM blebbistatin (Sigma-Aldrich, B0560) and plated on ultra-low attachment 6-well plates (Corning, 3471) (Day 0). On the next day, medium was changed to RPMI/B27-insulin supplemented with 5 µg/ml ascorbic acid, 10 ng/ml BMP4 and 25 ng/ml Activin A (Peprotech, 120-14E) (Day 1). On day 3, the medium was changed to differentiation medium RPMI/B27-insulin supplemented with 5 µg/ml ascorbic acid for 30 h. On the final step, medium was changed to RPMI/B27-insulin supplemented with 2.5 µM IWP-4. After 96 h, IWP-4 was removed from the media and medium was replaced with RPMI/B27 + insulin on day 11. After that, medium was refreshed three times a week. At day 17, the cardiomyocyte purity was improved by using magnetic activated cell sorting (MACS). First, MultiTissue Dissociation Kit 3 was used to dissociate beating aggregates to single cell level and after that, MACS was performed to dissociated cells according to kit instructions (both kits from Miltenyi Biotech, Germany). Directly after cell sorting, cardiomyocytes are used for establishment of co-cultures and multicultures.

### Development of hydrogel for 3D cell culture

#### Preparation of hydrazide-modified gelatin (Gelatin-CDH) and oxidized gellan gum (GGox) hydrogels

Gelatin was modified using carbodihydrazide (gela-CDH) as described previously (Koivisto et al. 2019). Briefly, 1000 mg of gelatin was dissolved in 500 ml water and kept at ambient temperature under N_2_ atmosphere. Then, 1.0 g (0.22 M) of CDH was added to this solution and the solution pH was adjusted to 4.7 with 0.5 M HCl. Parallel, 300 mg (0.04 M) of EDC (N,N-(3-dimethylaminopropyl)-N´-ethyl-carbodiimide hydrochloride) and 212 mg (0.03 M) of HOBt were dissolved in 3 mL DMSO/water (1.5:1 v/v) and added to the reaction mixture drop by drop, while keeping the solution pH at 6.8. The pH was monitored for 4 h and the reaction was kept under N2 atmosphere overnight. Then, the reaction product was precipitated in cold ethanol with NaCl (0.5 mg/mL). The precipitate was centrifuged, redissolved in water, and exhaustively dialyzed (12–14 kDa MWCO) and finally freeze-dried over 4 days for storage.

GG was modified by NaIO_4_ oxidation according to previously reported method (Karvinen et al. 2017) to produce GG-CHO with a modification degree of 25%. Briefly, 500 mg GG (Gelzan™ CM, Sigma Aldrich) was dissolved in 100 ml water at 90C and protected from light und N_2_ atmosphere. Then, 60 mg of NaIO_4_ were dissolved in water and added dropwise to the GG solution. The reaction was kept for 4h and then quenched using 300 µL ethylene glycol. The product was dialyzed (12–14 kDa MWCO) against water and freeze-dried over 4 days for storage.

### Preparation of hybrid hydrogel

To prepare the final hybrid hydrogel, Gelatin-CDH solution was dissolved in DMEM/F12 at 60 mg/ml and GGox at a concentration of 40 mg/ml. Both polymer solutions were filtered using a Whatman FP 30/0.2 CA-S sterile filter (Thermo Fisher Scientific) at + 37 ℃. The solutions were warmed + 37 ℃, and then equal volumes (1:1) of the solutions were mixed for a few seconds by pipetting to prepare the hydrogel directly for cell culture and as a hydrogel control.

### Mechanical characterization of the hydrogel

For rheological analysis of the hydrogel (Fig. S1), the polymers were dissolved as described above and tested using Discovery HR-2 rheometer with 20 mm plate-plate geometry and TRIOS software (TA Instruments). Appropriate values for amplitude (0.75% oscillation strain) and frequency (0.75 Hz) were known from previous measurements of similar materials and tests were carried out in triplicate. For the time sweep, 350µL GGox were placed on the bottom geometry at 37C, and the upper geometry was lowered to a gap of 1500 µm. While the upper geometry was spinning at 70 rad/s for 7 s, 350µL of Gela-CDH was added to gap so that the components would mix. The time sweep started immediately after the spinning step and was recorded for 1 h. Then, the hydrogel was left to rest for 5 min under the geometry, and an amplitude sweep from 0.01 to 100% oscillation strain (0.75 Hz) was performed on the same sample.

### Establishment of cell culture models

#### Cardiomyocyte 2D and 3D monocultures

**Fig. 1:**
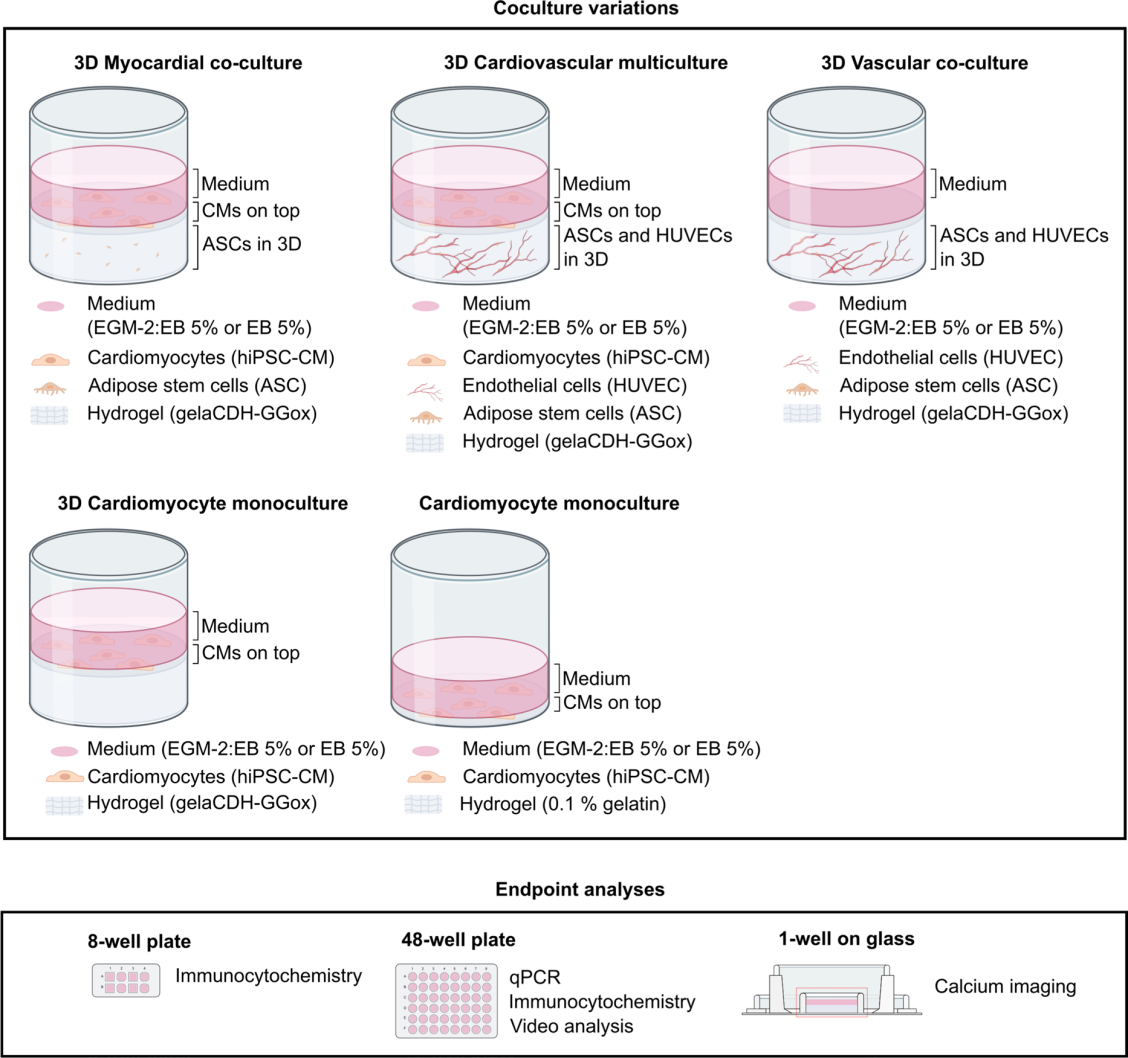
Composition of different cardiac models, co-culture variations and the endpoint analyses used in the study. Cardiomyocyte controls were plated as 2D monoculture on top of thin 0.1% gelatin coating or as 3D monoculture on top of gellan gum-gelatin hydrogel. Vascular co-cultures (ASC + EC), cardiovascular multiculture (ASC + CM + EC) and myocardial co-culture (ASC + CM) were formed in 3D in gellan gum-gelatin hydrogel

After MACS sorting, iPS-CM (UTA.04602.WT) were plated on top of thin, 0,1% gelatin (Sigma) coating as 2D monoculture or on top of gellan gum-gelatin hydrogel as 3D culture at 0.099 × 10^6^ cells/cm^2^ (Fig. 1) and cultured in 5% EB medium with KnockOut DMEM containing 5% FBS (Gibco and Biosera Nuaille), 1% NEAA, 1% Glutamax and 0.5% penicillin–streptomycin. 2D and 3D cardiomyocyte monocultures were imaged live during the culture period using EVOS microscope (EVOS FL Cell imaging system, Thermo Fisher Scientific) with brightfield view before end-point analyses.

#### 3D vascular co-cultures

Vascular network was formed through self-assembly of GFP-HUVEC and ASC in gellan gum-gelatin hydrogel in 48-well plates (Sigma-Aldrich) for qPCR analysis, 1-well on glass for calcium imaging, and in high content imaging 8-well plates (Ibidi) or in high content imaging 48-well plates (MatTek) for immunocytochemical staining or video analysis (Fig. 1). Briefly, ASC and GFP-HUVEC were detached and counted for cell ratio 1:5, respectively. ASC (p1 or p4) were carefully suspended into + 37 ℃ gelatin at 1 × 10^6^ cells/ml. Promptly, GFP-HUVEC (p3-5) were combined with gelatin-ASC suspension at 5 × 10^6^ cells/ml and finally + 37 ℃ gellan-gum was added to the suspension to start hydrogel crosslinking. The 3D co-cultures were left to gelate at + 37 °C for about 30 min before addition of angiogenic EGM-2 medium. EGM-2 medium was changed three times in a week to induce vascular network formation. After 7 days, EB 5% or EB 5% + EGM2 (1:1) medium was changed to vascular co-cultures before seeding of iPSC-CM or used as a 3D vascular control with EGM-2 medium. Vascular 3D co-cultures were imaged live during the culture period using EVOS microscope with brightfield view and GFP filter before end-point analyses.

#### 3D myocardial co-cultures

Myocardial 3D monocultures were established in 48-well plates (Sigma-Aldrich) for qPCR analysis, 1-well on glass for calcium imaging, and in high content imaging 8-well plates (Ibidi) or in high content imaging 48-well plates (MatTek) for immunocytochemical staining or video analysis (Fig. 1). Briefly, ASC (p1 or p4) were detached, counted, and carefully suspended into + 37 ℃ gelatin at 1 × 10^6^ cells/ml. Promptly, + 37 ℃ gellan-gum was added to the suspension to start hydrogel crosslinking. The 3D monoculture was left to gelate at + 37 °C for about 30 min before addition of EGM-2 medium. EGM-2 medium was changed three times in a week. After 7 days, EB 5% or EB 5% + EGM2 (1:1) medium was changed to ASC monocultures and iPSC-CM were seeded on top of the hydrogel at 0.099 × 10^6^ cells/cm^2^. Myocardial 3D co-cultures were imaged live during the culture period using EVOS microscope with brightfield view before end-point analyses.

#### 3D cardiovascular multicultures

After inducing vascular network formation for 7 days, EB 5% or EB 5% + EGM2 (1:1) medium was changed to vascular 3D co-cultures and iPSC-CM were seeded on top of the hydrogel at 0.099 × 10^6^ cells/cm^2^ (Fig. 1). Cardiovascular 3D multicultures were live imaged live during the culture period using EVOS microscope with brightfield view and GFP filter before end-point analyses.

#### Immunocytochemistry and imaging

The immunostaining protocol was adapted from Honkamäki et al. (2021). Briefly, after 16 days of culture, cells were washed with dPBS and fixed with 4% paraformaldehyde (Sigma-Aldrich) in dPBS for 60 min at RT. After three washes with dPBS, non-specific binding was blocked with solution containing 10% normal donkey serum (NDS, Millipore), 1% bovine serum albumin (BSA; Sigma-Aldrich) and 0.1% Triton X-100 (Sigma-Aldrich) in dPBS for 90 min at RT. Before primary antibody staining, samples were washed with solution containing 1% NDS, 0.1% TritonX-100 and 1% BSA in dPBS. We used primary antibodies against alpha smooth muscle actin (αSMA, Abcam, ab7817; 1:200) to identify the presence of smooth muscle cells and cardiac troponin t (Abcam, ab64623, 1:1500) to identify cardiomyocytes. Endothelial cells were visible due to of GFP (green fluorescent protein) expression in HUVEC. Primary antibodies were diluted in dPBS with 1% NDS, 1% BSA, 0.1% Triton X-100 solution and incubated for 3 days at 4 ℃. The samples were then washed for three times with 1% BSA in dPBS over a period of 2 days in total. Alexa Fluor 488 (A21202, 1:1500), 568 (A11057 and A11031 1:500 or 1:800) and 647 (A31571, 1:200) secondary antibodies (all from Thermo Fisher) in dPBS with 1% BSA were applied overnight at 4 ℃. The samples were washed with dPBS three times, stained with 0.2 µg/ml DAPI for 3 h at RT and finally washed three times with dPBS before imaging.

Imaging was mainly performed with Nikon Eclipse FN1 Upright Spinning Disk Confocal Microscope using Nikon Plan Fluor 10 × /0.3, WD 16.0 mm (Air)objective and using NIS Elements AR 5.11.00 imaging software (all from Nikon Corporation). Upright microscopy is used due to the thickness of the 3D samples. In addition, EVOS microscope was used to image GFP labeled vascular network and Leica Stellaris 8 confocal microscope with 10X dry objective (Leica Camera AG) to image cardiomyocyte microstructures. Images were further processed with ImageJ v.1.53f51 (Schindelin et al. 2012).

#### Quantitative real-time PCR

Before RNA collection, 3D cell culture samples were treated with non-specific protease pronase (Millipore) to break down the 3D structure. After pronase treatment, total RNA was extracted using Monarch^®^ Total RNA Miniprep Kit (New England Biolabs GmbH) and further converted to complementary DNA using High-Capacity cDNA Reverse Transcription Kit (Thermo Fisher) according to manufacturer´s protocols. The concentration and purity of the isolated RNA was measured with spectrophotometer (Nanodrop 2000; Thermo Fisher). Quantitative real time reverse transcription polymerase chain reaction (qRT-PCR) was performed with TaqMan Fast Advanced Master Mix (Thermo Fisher) and TaqMan Gene Expression Assays (Thermo Fisher, Table S3) according to manufacturers’ instructions. All measurements were performed with technical triplicates using QuantStudio 12 K Flex Real-Time PCR System (Applied Biosystems). *EEF1A1* (Elongation factor 1-alpha 1) and *GAPDH* (glyceraldehyde-3-phosphate dehydrogenase) were used as endogenous control genes for normalizing qRT-PCR data. Relative quantification of gene expression was performed using the 2^−ΔΔCt^ method (Livak and Schmittgen 2001).

### Functional analyses

#### Calcium imaging

Functional cardiac parameters from cardiovascular multicultures and myocardial co-cultures in EB5%:EGM-2 mixture medium were assessed using calcium imaging. Mixture medium was used due to supportive effect to both angiogenic and cardiac specific functions. Cells were loaded with 4 µM Fluo-4 AM (Thermo Fisher Scientific, Waltham, Massachusetts, USA) for 30 min in a perfusate medium. Perfusate medium consisted of 137 mM NaCl, 5 mM KCl, 1.2 mM MgCl2, 0.44 mM KH2PO4, 4.2 mM NaHCO3, 2 mM CaCl2, 1 mM Na pyruvate, 5 mM d-glucose, and 20 mM HEPES dissolved in distilled water (pH adjusted to 7.4 with NaOH). Sample chamber (Fig. 1) was placed onto a holder within Axio Observer.A1 microscope (Carl Zeiss, Germany) with heating plate preheated to 37 ℃ by a TC02-temperature controller (Multichannel Systems, Germany). Medium was changed to preheated medium before recording.

Ca^2+^ handling of spontaneously beating CM was recorded before and 3–5 min after administration of 1µM adrenaline (E4642, lot. MKCB911, Sigma-Aldrich) using UApo/340 × 20 air objective (Olympus Corporation, Tokyo, Japan) and ANDOR iXon 885 CCD camera (Andor Technology, Belfast, Northern Ireland) synchronized with a Polychrome V light source by a real-time DPS control unit. Fluo-4 AM was excited at 340 nm and 380 nm light and the emission was recorded with ZEN 2.0 (Carl Zeiss, Germany) for 30 s at 505 nm. Calcium imaging analysis was carried out by selecting regions of interest (ROIs) as single-beating iPSC-CM and the subtraction of background noise, recorded from a cell-free area in the same ROI, was done before further analysis.

#### Video microscopy

Functional cardiac parameters from multicultures and myocardial co-cultures were assessed using video microscopy. Monochrome videos were recorded for 30–60 s (720 × 480 resolution, 30 fps) using video microscopy (Nikon Eclipse TS100, Nikon Corporation, Tokyo, Japan) with a video camera Optika Digi-12 (Optika Microscopes, Ponteranica, Italy). Videos were analyzed using digital image-based correlation (DIC) based analysis method in custom-made software (Ahola et al. 2014) in MatlabR2019a (MathWorks, Inc., USA). Out of the signals obtained from a spontaneously beating iPSC-CM, average parameters of beating were defined as: (1) duration of contraction, (2) time when contracted, and (3) duration of relaxation.

### Statistical analysis

The data collected from iPSC-CM in cardiovascular multicultures and myocardial co-cultures and iPSC-CM from 3D controls iPSC-CM and their control iPSC-CM at the primary endpoint were pooled, respectively. Between data, standard error of mean (SEM) will be shown in the gene expression, cellular structure, and functional experiments. The significance between two groups (myocardial construct and cardiovascular construct) was evaluated with the unpaired Student’s *t* test. In functional experiments n refers to the number of cells. A p value less than 0.05 was considered statistically significant. The statistical significance of the functional and qPCR data was assessed by IBM SPPS Statistics 27-software by using Kruskal–Wallis test and with Bonferroni correction.

### Data availability

The data sets generated and analyzed for the current study are available from the corresponding author on reasonable request.

## Results

### Gellan gum-gelatin hydrogel supports formation of vascular network, smooth muscle cell-like network and elongated cardiomyocyte morphology in cardiac models

**Fig. 2:**
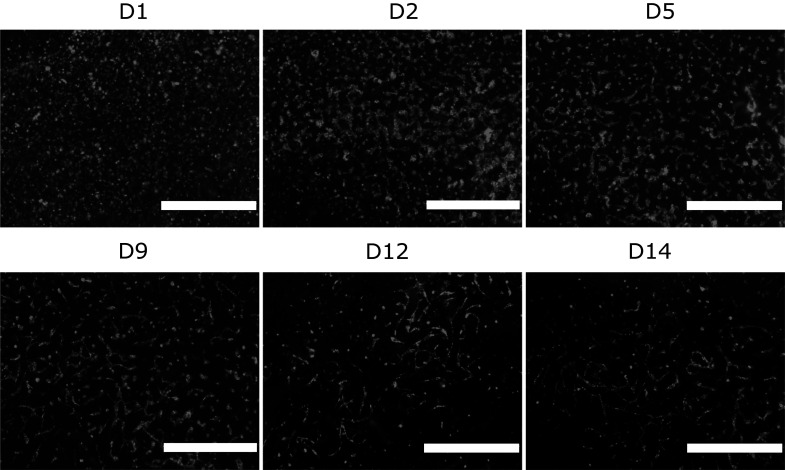
Progressive organization (d1-d14) of green fluorescent protein tagged endothelial cells into vascular network in 3D vascular co-culture formed by Human Umbilical Vein Endothelial Cells (HUVEC) and adipose tissue-derived stem/stromal cells (ASC). Scale bar 1 mm

Myocardial co-culture with ASC + CM and cardiovascular multiculture with ASC + CM + EC was established in gellan gum-gelatin hydrogel for 16 day culture in two different media. Cardiac (EB 5%) and 50:50 mixture of angiogenic and cardiac media (EB 5%:EGM-2) were used to support cardiomyocyte functionality and angiogenesis in the co- and multicultures. We first assessed the formation of vascular network by EC + ASC embedded in gellan gum-gelatin hydrogel in EGM-2 medium without CM and detected a progressive organization of GFP signaling EC into vascular network. After d9, gradual degradation of the vasculature was detected yet the connections between vessels were mainly remained throughout the culture (Fig. 2).

**Fig. 3:**
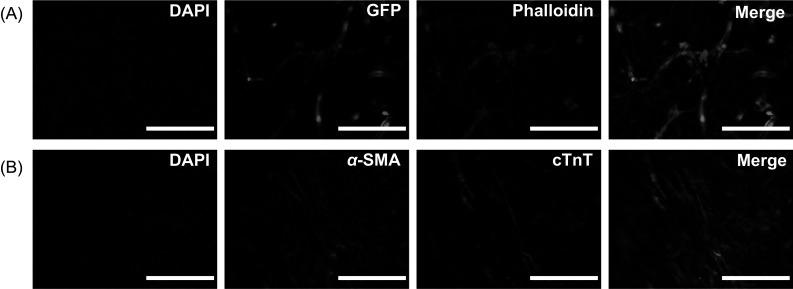
Formation of alpha-smooth muscle cell -positive cell network **A** in cardiovascular multiculture in EB 5% + EGM2 (1:1) medium at d9 shown with phalloidin staining and **B** in cardiovascular multiculture in EB 5% medium at d15 shown with α-alpha smooth muscle actin staining describe the alignment of cellular structures. Scale bar 1 mm

**Fig. 4:**
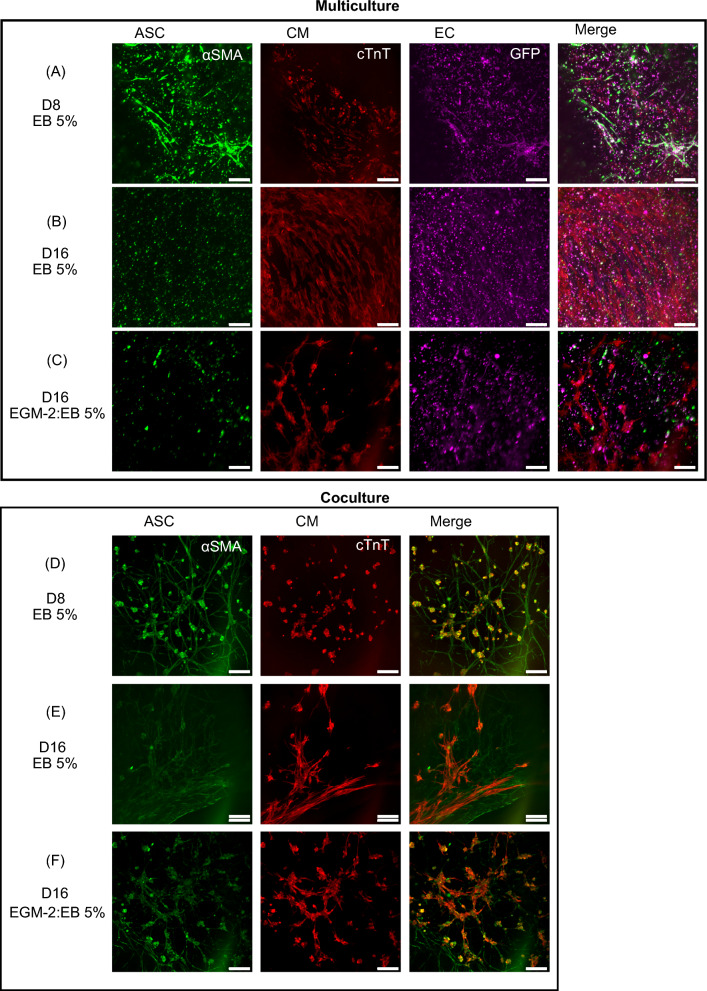
Formation of **A**–**C** cardiovascular multiculture comprising of human adipose stem/stromal cells (ASC), induced pluripotent stem cell-derived cardiomyocytes (iPSC-CM) and Human Umbilical Vein Endothelial Cells (HUVEC) at day 8 in EB 5% and at d16 in EB5%:EGM-2 and in EB 5%; **D**–**F** myocardial co-culture comprising of ASC and iPSC-CM in EB 5% (d8) and in EB 5%:EGM-2 and EB5% (d16) medium. ASC organize into smooth muscle cell-like network from d8 onwards as shown with α-smooth muscle actin staining (**D**–**F**). iPSC-CM shift into elongated morphology in both cardiac models as shown with α-troponin t staining (**A**–**F**). Vascular network formed by GFP signaling HUVEC degrades from d8 to d16 in multicellular construct (**A**–**C**). Scale bar 200 µm

We then established cardiovascular multiculture in EGM-2 to promote spontaneous self-assembly of EC and supporting ASC into vascular network for 7 days following a plating of CM in gellan gum-gelatin hydrogel. Figure 4 shows the organization of multicellular culture after plating of CM (d8) in EB 5% medium until the end-point (d16) in EB 5% or EB 5%:EGM-2 media. Before plating of CM, we detected an organization of two different cellular networks. At day 8, GFP signal from EC confirmed the organization of cells into partly connected vascular network (Fig. 4A). Gradual degradation of the formed vascular network was seen in cardiovascular multiculture in both media (Fig. 4A–C) similarly to vascular co-cultures. Additionally, interconnected cellular network independent from the vasculature was detected and stained positive with α-alpha smooth muscle actin (Fig. 3A, B). Moreover, the two network systems were partly aligned (Fig. S2). After plating of CM, media was changed either to EB 5% or to EB 5%:EGM-2. At day 8, CM appear rounded and randomly orientated in the multicellular culture (Fig. 4A). A clear change in CM morphology was detected during the multicellular culture as shown at day 16 with anisotropic, rod-like morphology of CM in both media (Fig. 4B, C). We detected also partial alignment of vascular network with the CM in cardiovascular multiculture (Fig. 4A–C).

Beside cardiovascular multiculture, we assessed the effect of ASC alone in culture with CM. We detected an organization of narrow, interconnected cellular network with elongated ASC that stained positive with α-alpha smooth muscle actin (Fig. 4D, F) and was distributed throughout the hydrogel area. The smooth muscle cell-like network remained intact throughout the 16-day culture period. While the CM remained rounded at day 8 (Fig. 4D), we saw a gradual shift in CM morphology in culture with ASC as shown at day 16 with anisotropic, rod-like morphology in both media (Fig. 4E, F). At day 16 in co-culture, elongated ASC were overlapping with cTnT positive CM in aligned structure (Fig. 4E, F).

Rheology was used to investigate the gelation and mechanical properties of the cell-free hydrogel (Fig. S1). The time sweep captures the gelation upon mixing of the two components and shows the gradual network development to a final storage modulus of 56 ± 19 kPa. The storage modulus (G’) increases more drastically than the loss modulus (G’’) as the hydrogel develops true-gel character. Clearly, the gelation still continues past the measurement time of 1h and the gel continues to set, but around approximately 30 min a transient point has been reached and we assume the hydrogel to be gelated. The amplitude sweep indicates the elastic nature of the hydrogel, as both moduli are well within the linear viscoelastic region until at least 80% oscillation strain. Finally, we followed the stability of cell-free gellan gum-gelatin hydrogels during the cell culture. Cell-free hydrogel controls were seen to gradually degrade (Fig. S3).

### Cardiomyocytes migrate into the hydrogel and form large and strongly beating areas

**Fig. 5:**
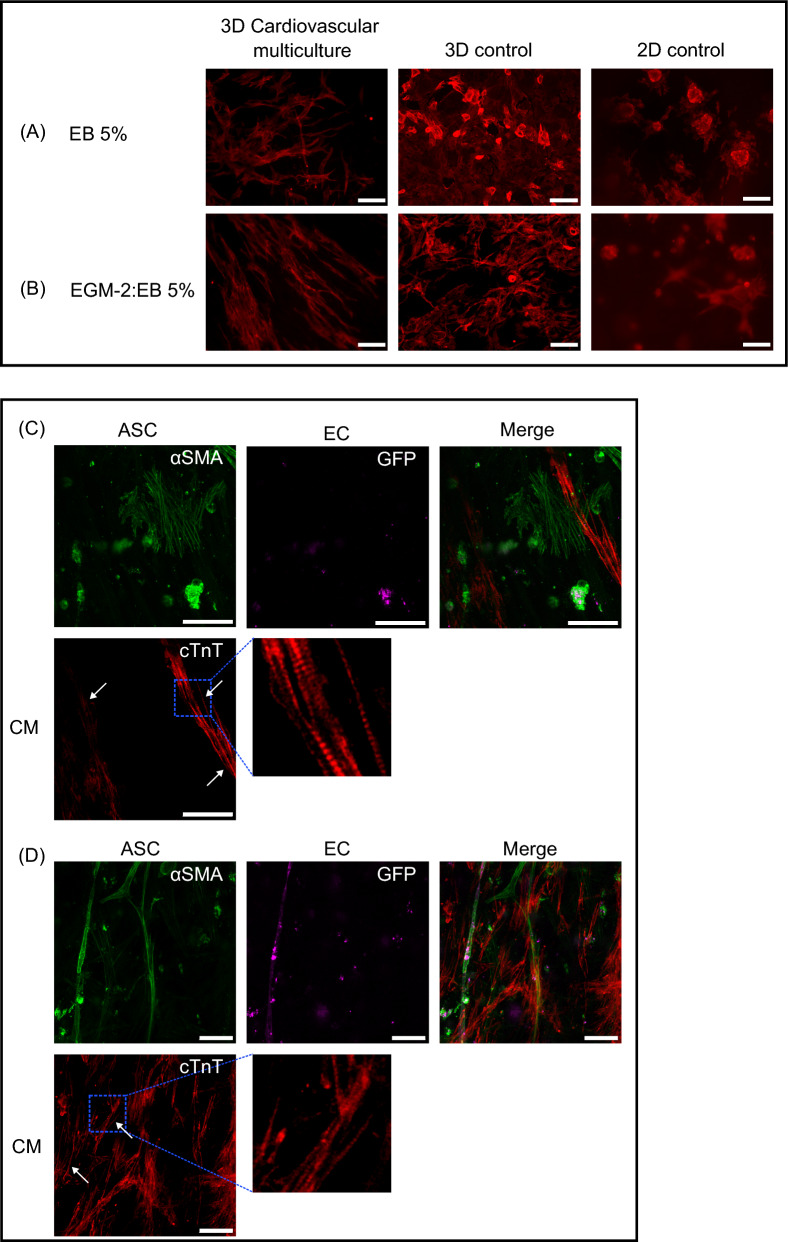
The morphology of CM in multicellular culture changes substantially when compared to 2D or 3D CM monocultures independently of the used media. 3D cardiovascular multiculture, 3D CM control and 2D CM control in **A** EB5% medium and **B** EGM-2:EB5% medium. Scale bar 200 µm. Microstructure of CM in the multiculture are presented in **C** and **D** with white arrows and insets pointing to the sarcomere structures. Scale bar 50 um

We detected CM migration from the hydrogel surface towards the other cell types residing inside the hydrogel block (Online Resource 1). In multi- and co-cultures, we detected exceptionally long and narrow, unilaterally oriented CM compared to control CM monocultures in 2D or in 3D environment. The change in CM morphology was not media dependent as morphological transition was detected in EB 5% as well as in 50:50 mixture media (Fig. 5A, B). Video analysis shows that contracting CM pulls the surrounding hydrogel in a synchronized manner (Online Resource 2). Furthermore, high-resolution microscopy showed repeating sections of sarcomeres in CM in multicellular culture (Fig. 5C, D).

### Myocardial co-culture improves contraction and calcium dynamics of cardiomyocytes

**Fig. 6:**
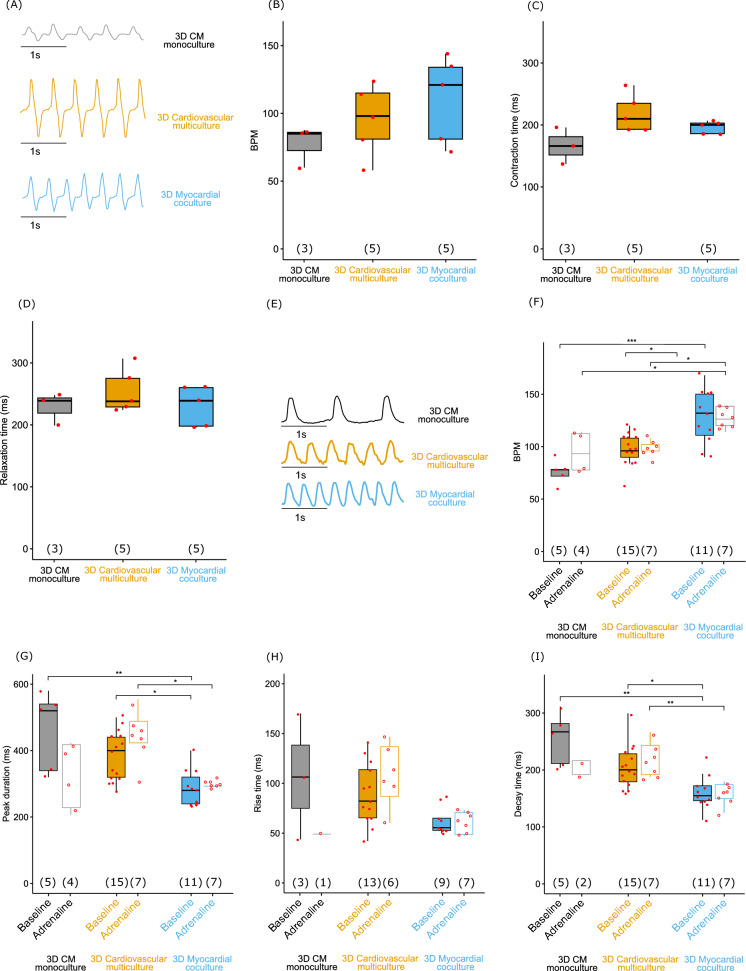
Representative beating traces of CM in 3D monoculture, cardiovascular multiculture and myocardial co-culture (**A**). Video microscopy analysis of (**B**) beating rate (Beats Per Minute, BPM), **C** duration of contraction and **D** relaxation time of 3D CM monoculture (control), cardiovascular multiculture, and myocardial co-culture in EGM-2:EB 5% medium at day 14 did not show statistically significant differences between the groups. Ca^2+^ transients analyzed from 3D CM monoculture (control), cardiovascular multiculture, and myocardial co-culture in EGM-2:EB 5% medium at day 14 baseline and after adrenaline exposure. Representative calcium traces at baseline (**E**). Data is represented as beating rate (BPM) (**F**), peak duration (**G**), rise- (**H**) and decay time (**I**). * *p* < 0.05, ** *p* < 0.01, *** *p* < 0.001; Kruskal–Wallis test with Bonferroni correction. Numbers in parentheses represent the number of cells used

To examine functionality of CM in cardiovascular multiculture (CM + ASC + HUVEC) and in myocardial co-culture (CM + ASC) in EGM-2:EB 5% medium, we studied contraction of the CM with video microscopy at day 14. Representative contraction traces can be seen in Fig. 6A. 3D CM monoculture in EB 5% medium was used as a control. When contraction was analyzed from the videos, the mean beats per minute (BPM) for 3D CM monoculture and cardiovascular multiculture presented physiologically normal resting heart rate (Bombardini et al. 2008), with higher frequency for myocardial co-culture. The mean BPM with 77.3 ± 9.6 for 3D CM monoculture, and 95.0 ± 11.7 for 3D cardiovascular multiculture was detected (Fig. 6B). Highest frequency with 110.4 ± 14.4 BPM was detected for 3D Myocardial co-culture. The mean contraction duration was 166.3 ± 17.0 ms for 3D CM monoculture, 218.8 ± 13.7 ms for 3D cardiovascular multiculture and 196.2 ± 4.5 ms for 3D myocardial co-culture (Fig. 6C). The mean relaxation time was 228.7 ± 15.1 for 3D CM monoculture, 254.6 ± 15.9 for 3D cardiovascular multiculture and 230.8 ± 14.4 for 3D myocardial co-culture (Fig. 6D). Statistically significant differences were not found in BPM, contraction time, nor in relaxation time of CM at day 14 in multi- or in co-cultures.

Next, we analyzed the functionality of CM by measuring calcium transients. Representative calcium traces can be seen in Fig. 6E. We detected an overall trend with more mature calcium dynamics in 3D myocardial co-culture producing strong and high frequency Ca^2+^ signals (Fig. 6F–I). During adrenergic stimulation, BPM (Fig. 6F) was higher in 3D myocardial co-culture along with shorter peak duration (Fig. 6G) and decay time (Fig. 6I). The mean BPM in 3D myocardial co-culture was 128.2 ± 7.8 ms at baseline (*p* < 0.0001 vs. 3D CM monoculture 75.6 ± 4.9 ms; *ns*. vs. 3D cardiovascular multiculture 98.0 ± 4.0 ms) and 126.9 ± 3.6 ms during 1µM adrenaline exposure (*p* < 0.05 vs. 3D CM monoculture 94.5 ± 9.6 ms and 3D cardiovascular multiculture 97.7 ± 2.8 ms). The mean peak duration in 3D myocardial co-culture was 285.5 ± 16.4 ms (*p* < 0.01 vs. 3D CM monoculture 460.0 ± 54.0 ms; *p* < 0.05 vs. 3D cardiovascular multiculture 384.0 ± 18.8 ms) during baseline and 300.0 ± 4.4 ms during 1µM adrenaline exposure (*ns*. vs. 3D CM monoculture 335.0 ± 46.5 ms; *p* < 0.05 vs. 3D cardiovascular multiculture 440.0 ± 27.6 ms). The mean rise time in 3D myocardial co-culture was 62.8 ± 4.6 ms during baseline (*ns.* vs. 3D CM monoculture 106.7 ± 36.5 ms; *ns*. vs. 3D cardiovascular multiculture 89.4 ± 36.5 ms) and 61.9 ± 3.8 ms during 1µM adrenaline exposure (*ns.* vs. 3D CM monoculture 50.2 ± 0 ms; *ns*. vs. 3D cardiovascular multiculture 109.6 ± 12.4 ms). The mean decay time in 3D myocardial co-culture was 161.4 ± 8.9 ms during baseline (*p* < 0.01 vs. 3D CM monoculture 255.0 ± 20.6 ms; *p* < 0.05 vs. 3D cardiovascular multiculture 216.8 ± 10.8 ms) and 156.8 ± 6.9 ms during 1µM adrenaline exposure (*ns.* vs. 3D CM monoculture 202.0 ± 12.1 ms; *p* < 0.05 vs. 3D cardiovascular multiculture 216.8 ± 10.8 ms).

Representative video recording of the calcium transient of cardiovascular multiculture can be seen in Online Resource 3 showing strong contraction of the CM accompanied with calcium transient. Notable larger 3D CM monoculture calcium transient amplitude (data not shown) can be seen in the Online Resource 4. In 2D control, synchronous calcium release (data not shown) can be seen on single CM (Online Resource 5).

### Gene expression analysis shows constant expression of cardiac and angiogenesis related genes

Expression levels of angiogenesis and cardiac related genes were analyzed from vascular 3D co-cultures, cardiovascular multicultures and from myocardial co-cultures to study formation of vascular and smooth muscle cell-like networks and to assess the maturation state of CM. Structure-related cardiac genes *troponin T* (*TNNT2*) and *connexin 43* (*GJA1*) were analyzed from cardiovascular multicultures at day 1 and 16 and from myocardial co-cultures at day 16 (Fig. S4) In cardiovascular multiculture and in myocardial co-culture, we detected constant *TNNT2* expression in all time points. Similarly, *GJA1* expression remained constant.

With angiogenesis-related genes, we studied the expression of angiogenic growth factors and α-SMA at day 1 and 16 in vascular 3D co-cultures and in cardiovascular multicultures and at day 16 in myocardial co-cultures (Fig. S4). We detected an increasing trend in the expression of *fibroblast growth factor 2* (*FGF-2*) in vascular 3D co-culture whereas the expression remained constant in cardiovascular multiculture and decreased in myocardial co-culture. Similarly, the expression of *alpha smooth muscle actin* (*ACTA*) showed an increasing trend in vascular 3D co-culture during the culture period whereas it remained constant in cardiovascular multiculture and decreased in myocardial co-culture. Expression of another angiogenic growth factor *vascular endothelial growth factor A* (*VEGF-A*) showed slightly increasing trend in vascular 3D co-culture from day 1 to day 16. Interestingly, after plating of CM in cardiovascular multiculture at day 8, increase in *VEGF* was detected following a decrease until day 16. Modest *VEGF-A* expression was detected in myocardial co-culture at day 16. Another angiogenic marker, *angiopoietin 1* (*Ang 1*) showed an increasing expression trend in vascular 3D co-culture from day 1 to day 16 whereas it remained constant in cardiovascular multiculture. Myocardial co-culture showed modest expression of *Ang 1*. *Angiopoietin 2* (*Ang 2*) remained constant in vascular 3D co-culture but showed slightly decreasing trend in cardiovascular multiculture during culture. In myocardial co-culture, expression of *Ang 2* was not detected. Statistical differences between the groups or between different time points were not detected.

## Discussion

In vitro models are platforms to study normal cellular and molecular physiology as well as diseased cardiovascular physiology. Detailed information on the underlying disease mechanisms is needed to reveal the pathophysiology for various cardiovascular diseases and to provide new treatment options. Additionally, in vitro heart models are needed for screening of novel drug candidates, and for early cardiac safety and efficacy assessment. Currently, there is no universal in vitro model capable of recapitulating all heart tissue characteristics at structural, functional, and genetic level. Indeed, several in vitro cardiac models are needed to meet the needs for different cardiac applications, and developed model systems should truthfully mimic human responses. In order to resemble the human heart, the cardiac in vitro model should include 3D structure, and CM interactions with other cell types naturally present in heart (Vuorenpää et al. 2023).

Previously, we combined neonatal rat cardiomyocytes with vascular-like network to improve viability, maturation and functionality of CM (Vuorenpää et al. 2014). The model system was further improved with human iPSC- and ESC-derived cardiomyocytes to achieve human relevant in vitro cardiac model with adult CM characteristics (Vuorenpää et al. 2017). In the current study we moved from 2 to 3D environment and established myocardial co-culture with ASC + CM and cardiovascular multiculture with EC + ASC + CM to study some key cardiovascular characteristics and to reveal differences in CM functionality in two different cellular set-ups. We chose ASC in co- and multicultures since, despite the long-term utilization of mesenchymal stem cells in cardiovascular tissue engineering, their mode of action is still partly unknown. Mesenchymal stem cells are an attractive choice for tissue engineering due to their immunosuppressive properties, easy availability, fast in vitro expansion, and for their regenerative capacity (Lindroos et al. 2011; Patrikoski et al. 2014; Han et al. 2019). MSC also produce extensively ECM proteins (Kalinina et al. 2015) as well as angiogenic and anti-apoptotic factors (Rubina et al. 2009; Sarkanen et al. 2012; Matsuda et al. 2013). When co-cultured with EC, MSCs promote vascular network formation by functioning as pericytes (Vuorenpää et al. 2017; Mykuliak et al. 2022). Based on these earlier findings by us and others, we chose ASC and ASC supported vascularization to enhance CM functionality in 3D environment in the current study.

We first assessed the formation of vascular network by HUVEC + ASC in gellan gum-gelatin hydrogel. The hybrid hydrogel was chosen according to our previous result showing biomimicking elastic properties that are comparable with native heart tissue (Koivisto et al. 2019). In this study, we detected a progressive formation of vascular network until day 9 and gradual degradation after that. As intact, interconnected vascular network throughout the hydrogel area could not be retained, we conclude that gellan gum-gelatin hydrogel gives modest support for generation of vascular network in cardiovascular multiculture. To our surprise, we found that instead of extensive vascular network, the hydrogel seems to support organization of ASC into alpha smooth muscle actin-positive cellular network. Although differentiation of ASC into smooth muscle cells was not verified during the study, a major shift in morphology towards elongated, alpha smooth muscle actin-positive phenotype in addition to organization into densely connected network implies an ongoing differentiation process. These findings are in line with our recently published results comparing different bioactive gellan gum-based hydrogels in in vitro and in vivo applications (Gering et al. 2022). Although Silva-Correia et al. similarly reported lack of angiogenic support by their gellan gum-based hydrogels (Silva-Correia et al. 2012), other gellan gum-based modifications have been successfully used to support vascularization (Cerqueira et al. 2014; Chen et al. 2018). Suitable elasticity is required for the encapsulating hydrogel to allow the cells to modify their microenvironment instead of being entrapped inside a rigid polymer network. We hypothesize that EC and supportive stromal cells might benefit with decreased hydrogel stiffness in vascularization. Indeed, in previous reports, gellan gum has been used for hard tissue engineering applications with some efforts to vascularize the target tissue (Oliveira et al. 2010; Vuornos et al. 2020). Decreasing the hydrogel stiffness might improve its suitability for soft tissue engineering and support vascular network maintenance.

We then analyzed CM structural properties in 3D cardiovascular multiculture (CM + ASC + EC) and in myocardial co-culture (CM + EC) using different microscopy techniques. In the cardiovascular multiculture, we detected a significant structural transition from round phenotype towards elongated, rectangular and aligned CM morphology. This is in line with earlier results in 2D environment by us (Vuorenpää et al. 2017) and others (Koivisto et al. 2022) but also in 3D multicellular constructs (Giacomelli et al. 2020). Anisotropic, rectangular shape of cardiomyocytes has been shown to facilitate myofibril alignment and contractility (Yang et al. 2014). In microstructural analysis of cardiovascular multiculture, we detected organized sarcomere development and alignment of CM with other cell types. Structural analyses suggest an improved functionality of CM in cardiovascular multiculture and in myocardial co-culture. Similar multicellular 3D cardiac microtissues established by Giacomelli et al. also showed that multiculture of iPSC-CM, fibroblasts and EC improved structural maturation of CM especially with cardiac-specific fibroblasts (Giacomelli et al. 2020). Yoshida et al. reported that CM in 2D environment showed significantly higher sphericity index, i.e. more round phenotype in CM monocultures compared to bone marrow stem/stromal cell-CM co-cultures (Yoshida et al. 2018). Additionally, they showed that CM had significantly lower average cell size in monocultures when compared to co-cultures similarly to us. In 3D co-culture of iPSC-CM and iPSC-fibroblasts in fibrin matrix, de Lange et al. detected presence of t-tubular system after two weeks of culture as an indication of functional mature cardiac tissue (de Lange et al. 2021). Based on microscopical analysis on CM structural changes in our study, we can conclude that cardiovascular multiculture as well as myocardial co-culture support CM transition towards adult phenotype.

Our functional video analysis of cardiovascular multiculture and myocardial co-culture supported the structural analysis and showed increased contractility and promotion of electrophysiological development of CM similarly to Yoshida et al., (2018). Additionally, we analyzed the calcium transients in myocardial co-cultures and detected increased beating frequency and shorter calcium transient time compared to CM monoculture, which commonly have poorly established calcium dynamics (Hwang et al. 2015; Koivumäki et al. 2018). Addition of adrenaline did not give expected effect on BPM, except with CM monoculture, which can be a limitation in our experimental setup as adrenaline cannot diffuse fast enough into the hydrogel in cardiovascular multiculture and in myocardial co-culture. Gene expression analysis revealed a trend in the expression of angiogenesis and cardiac related genes supporting the findings from structural and functional analyses of our study.

We visually detected gradual degradation of cell-free hydrogels during 16-day culture period. Interestingly, inclusion of cells, especially ASC, seemed to preserve the gellan gum-gelatin hydrogel when compared to the cell-free controls, as detected by daily visual inspection. Alpha smooth muscle cell-like network that spread throughout the hydrogel area seemed to retain the 3D structure coherent yet flexible despite the strong pumping movement by contracting CM. ASC are known to produce variety of ECM proteins and modify their microenvironment (Kalinina et al. 2015). We speculate that ECM production beside the smooth muscle cell-like network might contribute to the hydrogel integrity with positive effect to CM contractility.

To summarize, we established two human cell-based cardiac models in gellan gum-gelatin hydrogel and assessed the functionality of CM in the models. We demonstrated that cardiovascular multiculture composed of ASC + CM + EC and myocardial co-culture with ASC + CM support CM functionality in gellan gum-gelatin hydrogel. We detected formation of two different network systems with more extensive organization of smooth muscle cell-like network compared to vascular network in both cellular set-ups. Our study demonstrated the importance of microenvironment for different cell types and produced two novel cardiac models that can be utilized in cardiovascular research.

### Supplementary Information

Below is the link to the electronic supplementary material.

Supplementary file1 (MP4 5983 KB)

Supplementary file2 (MP4 5578 KB)

Supplementary file3 (MP4 4638 KB)

Supplementary file4 (MP4 4685 KB)

Supplementary file5 (MP4 4694 KB)

Supplementary file6 (EPS 166 KB)

Supplementary file7 (EPS 24210 KB)

Supplementary file8 (EPS 2663 KB)

Supplementary file9 (EPS 146 KB)

Supplementary file10 (EPS 184 KB)

Supplementary file11 (DOCX 3501 KB)
