# Supplementary material for: Gellan gum-gelatin based cardiac models support formation of cellular networks and functional cardiomyocytes
Source: Cytotechnology. 2024 May 2;76(4):483–502. doi: 10.1007/s10616-024-00630-5 (PMC11196475; doi:10.1007/s10616-024-00630-5)
Supplement: Supplementary file 11 — Supplementary file11 (DOCX 3501 KB) [file 10616_2024_630_MOESM11_ESM.docx]

Supplementary Material

Gellan gum-gelatin based cardiac models support formation of cellular networks and functional cardiomyocytes

**Hanna Vuorenpää^1,2*^, Joona Valtonen^3^, Kirsi Penttinen^3^, Sanna Koskimäki^1-3^, Emma Hovinen^1-3^, Antti Ahola^4^, Christine Gering^5^, Jenny Parraga^5^, Minna Kelloniemi^6^, Jari Hyttinen^4^, Minna Kellomäki^5^, Katriina Aalto-Setälä^3,7†^, Susanna Miettinen^1,2†^, Mari Pekkanen-Mattila^3†^**

^1^Adult Stem Cell Group, Faculty of Medicine and Health Technology, Tampere University, Tampere, Finland

^2^ Tays Research Services, Wellbeing Services County of Pirkanmaa, Tampere University Hospital, Tampere, Finland

^3^Heart Group, Faculty of Medicine and Health Technology, Tampere University, Tampere, Finland

^4^Computational Biophysics and Imaging Group, Faculty of Medicine and Health Technology, Tampere University, Tampere, Finland

^5^Biomaterials and Tissue Engineering Group, Faculty of Medicine and Health Technology, Tampere University, Tampere, Finland

^6^Department of Plastic and Reconstructive Surgery, Tampere University Hospital, Tampere, Finland

^7^Heart Hospital, Tampere University Hospital, Tampere, Finland

***** Correspondence: Hanna Vuorenpää, [hanna.vuorenpaa@tuni.fi](mailto:hanna.vuorenpaa@tuni.fi), ORCID 0000-0002-6351-3044

† these authors contributed equally

# Supplementary Tables

Table S1. Information on adipose tissue derived stem/stromal cell line (ASC) donors, cell passage and experiments performed in the study.

| **Donor ID** | **Gender** | **Age** | **Cell passage used** | **Experiment** |
| --- | --- | --- | --- | --- |
| **ASC 1** | **Male** | **56** | 1 | qPCR, video microscopy |
| **ASC 2** | **Female** | **31** | 1 | immunocytochemistry, calcium imaging, video microscopy |
| **ASC 3** | **Female** | **33** | 1, 4 | immunocytochemistry, calcium imaging, video microscopy |

Table S2. Surface marker expression of the studied donor ASC 1-3 lines. In the table, individual donor cell line passage during analysis is denoted in the column ‘’P’’. Positive > 80%; low < 10%; negative < 2%. The cells were identified as mesenchymal stem cells due to positive expression of CD73, CD90, and CD105, and low or negative expression of CD14, CD19 and CD45 (Dominici et al. 2006; Bourin et al. 2013). The expression of CD34 and HLA-DR was present at variable levels. The heterogeneity in surface marker expression if compared to the ISCT requirements could be explained by changes in cell culturing conditions (Patrikoski et al. 2013).

|  | | **Surface marker expression** | | | | | | | |
| --- | --- | --- | --- | --- | --- | --- | --- | --- | --- |
|  |  | **CD14**  low to negative | **CD19**  low to negative | **CD34**  variable | **CD45**  low to negative | **CD73**  positive | **CD90**  positive | **CD105**  positive | **HLA-DR**  variable |
| **Donor ID** | **P** |  |  |  |  |  |  |  |  |
| **ASC 1** | 2 | 0,5 | 0,3 | 37 | 0,7 | 96,8 | 98,1 | 99,8 | 1 |
| **ASC 2** | 2 | 7,1 | 7,7 | 11,5 | 8,7 | 99,5 | 99,8 | 100 | 1 |
| **ASC 3** | 2 | 0.9 | 0,8 | 51,3 | 3,4 | 93,3 | 99,4 | 99,8 | 1,2 |

Table S3. TaqMan Gene Expression Assays used in qPCR (Thermo Fisher)

| Gene​ | Protein​ | Taqman code​ |
| --- | --- | --- |
| *GJA1* | Connexin-43 | [Hs00748445_s1](https://www.thermofisher.com/taqman-gene-expression/product/Hs00748445_s1?CID=&ICID=&subtype=) |
| *TNNT2* | Cardiac type troponin T2 | Hs00165960_m1 |
| VEGF-A | Vascular endothelial growth factor A | Hs00900055_m1 |
| FGF-2​ | Fibroblast Growth Factor 2 | ​Hs00266645_m1 |
| Ang1 | Angiopoietin 1 | Hs00375822_m1 |
| Ang2​ | Angiopoietin 2​ | ​Hs00169867_m1 |
| ACTA | Alpha smooth muscle actin | Hs00426835_g1 |
| EEF1A1 | Elongation factor 1-alpha 1 | Hs00265885_g1 |
| (endogenous control) |  |  |
| TBP | TATA-box binding protein | Hs00427620_m1 |
| (endogenous control) |  |  |

# Supplemental figures


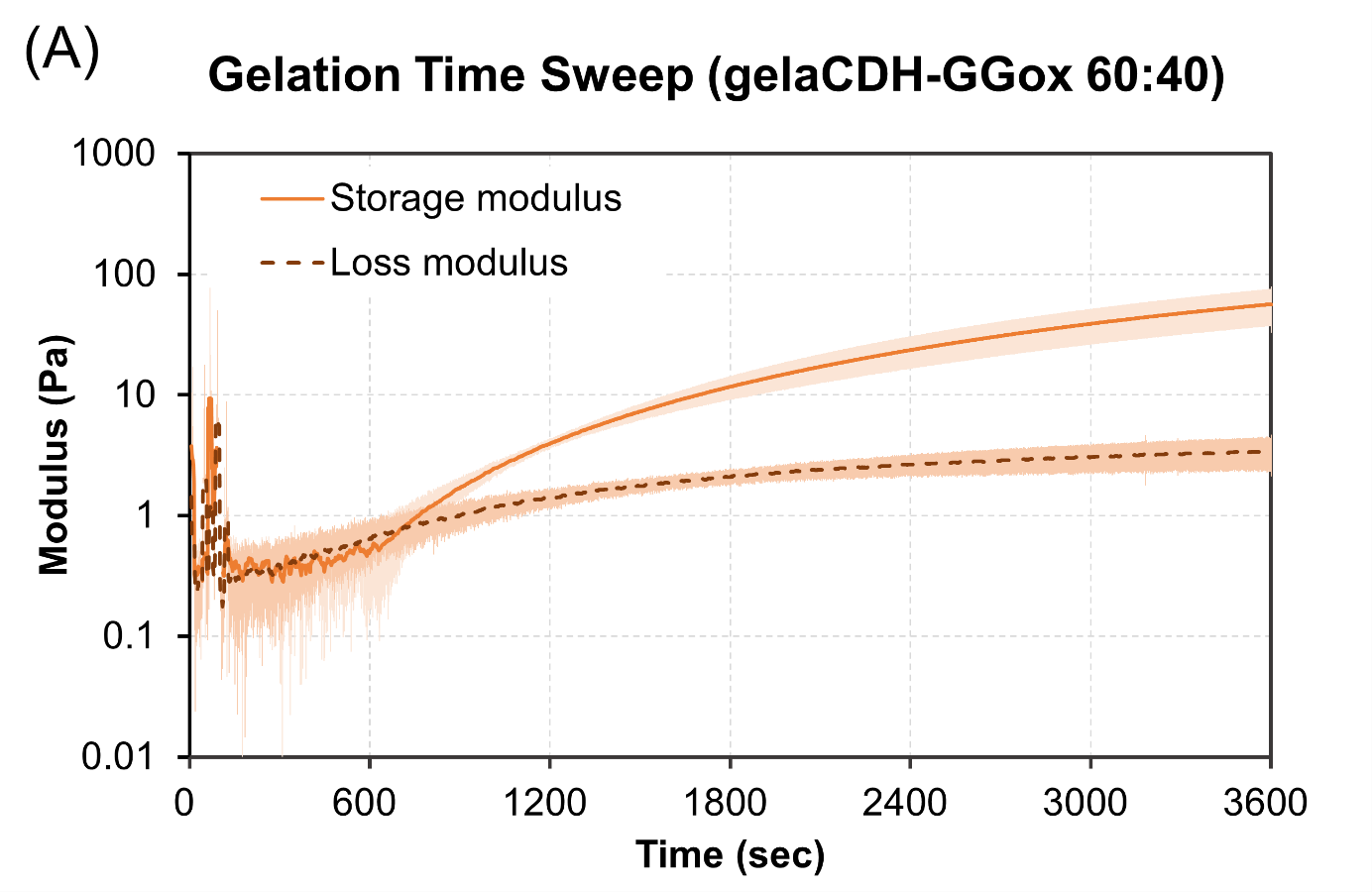

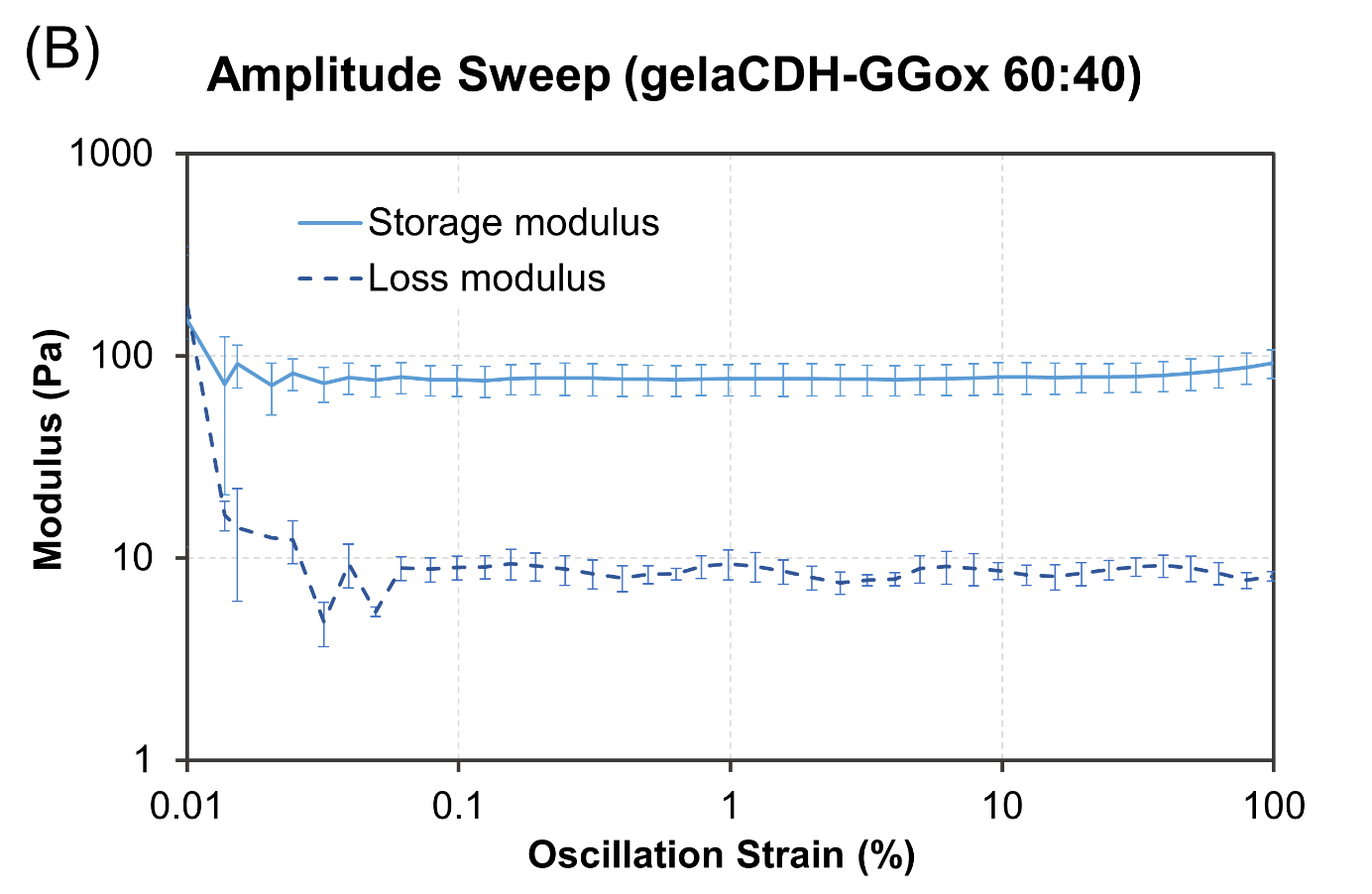


**Fig S1:** **Mechanical testing of the gelatin (gelaCDH) and gellan gum (GGox).** (A) Gelation time sweep, (B) Amplitude sweep. (n=3).


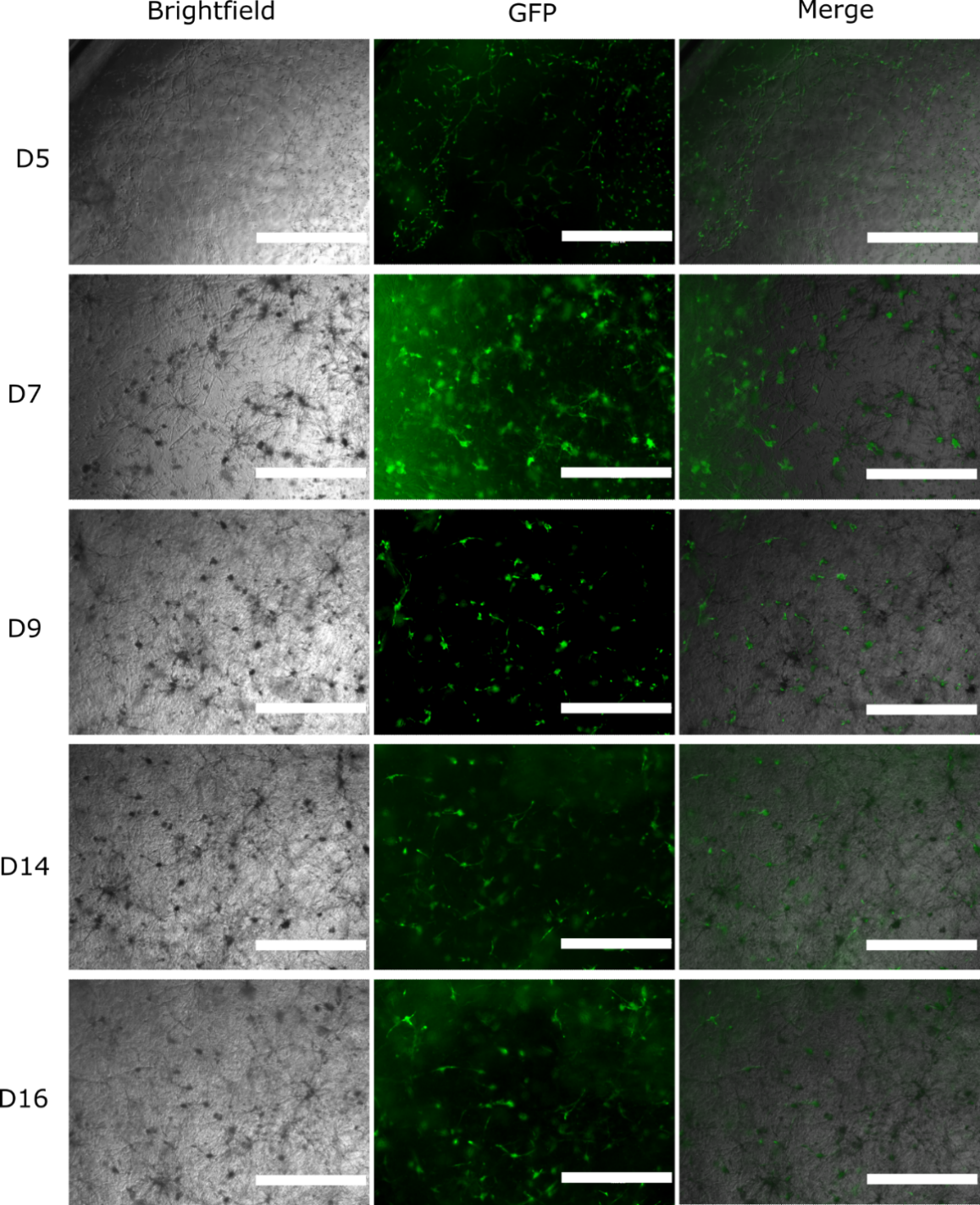


Fig S2: Formation and alignment of two different networks in cardiovascular multiculture. Smooth muscle cell-like network formed by ASC partly align with the vasculature (shown with GFP signal from EC) before plating of CM (d5-d7) and after plating of CM (d9-d16). Scale bar 1 mm.


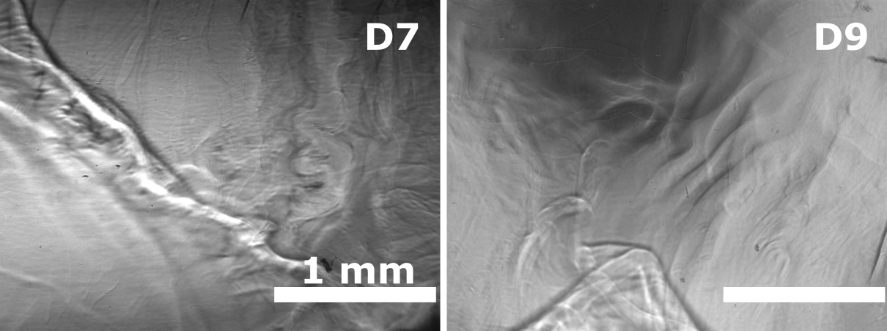


**Fig S3:** Cell-free hydrogel controls were seen to gradually degrade (d7, d9).


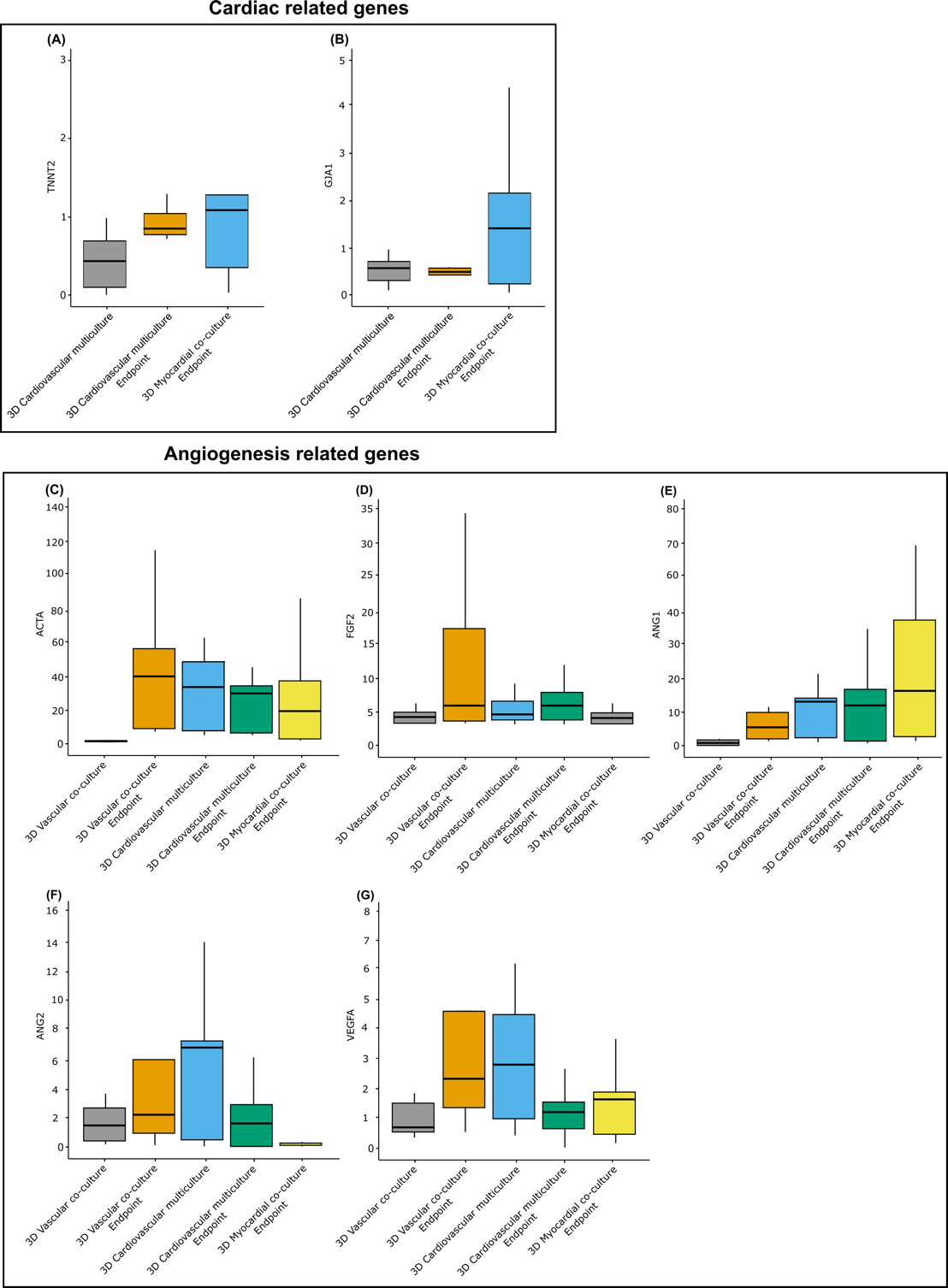


**Fig S4:** Expression levels of cardiac related genes (A-B) were analyzed from cardiovascular multicultures and from myocardial co-cultures at day 1 and 16 to study expression of structural genes for (A) *troponin T* (*TNNT2*) and (B) *connexin 43* (*GJA1*). Expression levels of angiogenesis related genes (C-G) were analyzed from vascular 3D co-cultures, cardiovascular multicultures and from myocardial co-cultures at day 1 and 16 to study expression of (C) *alpha smooth muscle actin* (*ACTA*)*,* (D) *fibroblast growth factor 2 (FGF-2)*, (E) *vascular endothelial growth factor A* (*VEGF-A*), (F) *angiopoietin 1* (*Ang 1*) and (G) *angiopoietin 2* (*Ang 2*).

# Online Resources

**Online Resource 1**. Representative video recording showing the CM migration from the hydrogel surface towards the other cell types residing inside the hydrogel block.

**Online Resource 2**. Representative video recording showing how CM pulls the surrounding hydrogel in a strong and synchronized manner.

**Online Resource 3**. Representative video recording of the calcium transient of 3D cardiovascular multiculture.

**Online Resource 4**. Representative video recording of the calcium transient of 3D CM monoculture showing notably larger Ca^2+^ transient amplitude.

**Online Resource 5**. Representative video recording of the calcium transient of 2D control showing synchronous Ca^2+^ release.

**References**

Bourin, P, Bunnell B.A, Casteilla L, et al (2013). Stromal Cells from the Adipose Tissue-Derived Stromal Vascular Fraction and Culture Expanded Adipose Tissue-Derived Stromal/Stem Cells: A Joint Statement of the International Federation for Adipose Therapeutics and Science (IFATS) and the International Society for Cellular Therapy (ISCT). *Cytotherapy* 15 (6): 641–48. DOI: [10.1016/j.jcyt.2013.02.006](https://doi.org/10.1016/j.jcyt.2013.02.006)

Dominici M, Le Blanc K, Mueller I, et al (2006). Minimal Criteria for Defining Multipotent Mesenchymal Stromal Cells. The International Society for Cellular Therapy Position Statement. *Cytotherapy* 8 (4): 315–17. DOI: [10.1080/14653240600855905](https://doi.org/10.1080/14653240600855905)

Patrikoski M, Juntunen M, Boucher S, et al (2013). Development of Fully Defined Xeno-Free Culture System for the Preparation and Propagation of Cell Therapy-Compliant Human Adipose Stem Cells. *Stem Cell Research & Therapy* 4 (2): 27. DOI: [10.1186/scrt175](https://doi.org/10.1186/scrt175) s
